# The permeability of SPION over an artificial three-layer membrane is enhanced by external magnetic field

**DOI:** 10.1186/1477-3155-4-4

**Published:** 2006-04-07

**Authors:** Fadee G Mondalek, Yuan Yuan Zhang, Bradley Kropp, Richard D Kopke, Xianxi Ge, Ronald L Jackson, Kenneth J Dormer

**Affiliations:** 1 Department of Chemical, Biological and Materials Engineering, University of Oklahoma, Norman, OK, USA; 2 Department of Urology, University of Oklahoma Health Sciences Center, Oklahoma City, OK, USA; 3 Hough Ear Institute, Oklahoma City, OK, USA; 4 Naval Medical Center, San Diego, CA, USA; 5 Department of Physiology, University of Oklahoma Health Sciences Center, Oklahoma City, OK, USA

## Abstract

**Background:**

Sensorineural hearing loss, a subset of all clinical hearing loss, may be correctable through the use of gene therapy. We are testing a delivery system of therapeutics through a 3 cell-layer round window membrane model (RWM model) that may provide an entry of drugs or genes to the inner ear. We designed an *in vitro *RWM model similar to the RWM (will be referred to throughout the paper as RWM model) to determine the feasibility of using superparamagnetic iron oxide (Fe_3_O_4_) nanoparticles (SPION) for targeted delivery of therapeutics to the inner ear.

The RWM model is a 3 cell-layer model with epithelial cells cultured on both sides of a small intestinal submucosal (SIS) matrix and fibroblasts seeded in between. Dextran encapsulated nanoparticle clusters 130 nm in diameter were pulled through the RWM model using permanent magnets with flux density 0.410 Tesla at the pole face. The SIS membranes were harvested at day 7 and then fixed in 4% paraformaldehyde. Transmission electron microscopy and fluorescence spectrophotometry were used to verify transepithelial transport of the SPION across the cell-culture model. Histological sections were examined for evidence of SPION toxicity, as well to generate a timeline of the position of the SPION at different times. SPION also were added to cells in culture to assess *in vitro *toxicity.

**Results:**

Transepithelial electrical resistance measurements confirmed epithelial confluence, as SPION crossed a membrane consisting of three co-cultured layers of cells, under the influence of a magnetic field. Micrographs showed SPION distributed throughout the membrane model, in between cell layers, and sometimes on the surface of cells. TEM verified that the SPION were pulled through the membrane into the culture well below. Fluorescence spectrophotometry quantified the number of SPION that went through the SIS membrane. SPION showed no toxicity to cells in culture.

**Conclusion:**

A three-cell layer model of the human round window membrane has been constructed. SPION have been magnetically transported through this model, allowing quantitative evaluation of prospective targeted drug or gene delivery through the RWM. Putative *in vivo *carrier superparamagnetic nanoparticles may be evaluated using this model.

## Background

Biocompatible magnetic micro and nanoparticles are being extensively studied by researchers worldwide for possible magnetically enhanced targeted delivery of therapeutics [1]. In these systems, therapeutics (e.g. drugs or genes) are attached to the magnetic particles and injected near the target site. A magnetic field is then applied to the site externally in order to concentrate the particles at the target site. In gene therapy applications, magnetic non-viral delivery systems have achieved promising results in transfection and expression rates without any immunogenic complications [2]. In the case of drug delivery, therapeutic drugs are concentrated at the site in the body where they are needed; thereby, reducing side effects and minimizing the required dose [3-5].

Due to their unique magnetic properties not found in other materials, magnetic nanoparticles have shown promising results in biomedical applications as well [1]. For example: data storage nanostructures (magnetic nanocrystal arrays) [6], biomedical applications, optoelectronics, smart imaging probes [7], biomedical nanostructure fluids, biodegradable microspheres [8], drug and gene delivery systems [9,10], biomagnetic separations [11], magnetic nanocomposites [12], magnetic fluid seals [13], hyperthermia cancer treatment [14] and magnetic synthesis [15].

Presently, several types of SPION are commercially available. They vary in size, magnetic properties and chemical composition (although the optimal ferrite is magnetite, Fe_3_O_4_). Depending on their size, SPION may exhibit a superparamagnetic state. In this case, particles exhibit no remanence in the absence of an external magnetic field. Any external magnetic force exerted on the particle is a translational force directed along the applied field vector and is dependent on the magnetic properties of the particle and the surrounding medium, the size and shape of the particles and the product of the magnetic flux density and the field gradient.

Deafness due to sensorineural injury might be correctable in hearing impaired patients. Gene therapy may be for hair cell loss in the future, but not for all the deafness. True restoration of hearing has not happened yet. Delivery of therapeutics to the inner ear is minimally successful today. Gene therapy for hearing disorders using viral vectors would likely present immunological complications and possible mutations. Recently, scientists were successful in restoring hearing to a mammal through adenoviral transfection of the Math1 gene [16]. The human RWM is about 70 μm thick and is made up of 3 layers [17-21]: an outer epithelium facing the middle ear, a core of connective tissue, and an inner epithelium that bounds the inner ear. Our goal is to design a minimally invasive delivery system for biological molecules to the inner ear through the RWM, an entry site to the inner ear cochlear fluids. Accordingly, we designed an *in vitro *RWM model to determine the feasibility of testing SPION for potential targeted delivery of therapeutics to the inner ear.

## Results

### Model Design

**Figure 1:**
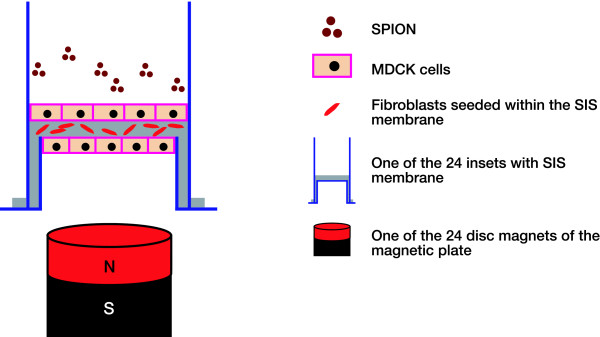
**The RWM Model Design**. A schematic representation of the RWM model and the magnetic delivery system.

We developed a 3 cell layer RWM model similar to the human RWM, consisting of two epithelial layers cultured on both side of a supporting collagen matrix, similar to the inner and outer epithelium layers in the human. The supporting matrix in the model was the small intestinal submucosa (SIS) that has a high density of collagen fibers and is seeded with human fibroblasts, again similar to the connective tissue middle layer in the actual RWM. The SIS membrane is a xenogenic porcine membrane harvested from the small intestine in which the tunica mucosa, serosa and tunica muscularis were physically removed from the inner and outer surfaces. The result is a collagen-rich membrane, approximately 80–100 μm thick and composed mainly of the submucosal layer of the intestinal wall [22]. The SIS membrane has two sides [23]: a serosal side facing the outside of the intestine and a mucosal side facing the intestinal lumen. The mucosal side is more permeable than the serosal side by seven-fold. Madin Darby Canine Kidney (MDCK) epithelial cells as well as human bladder urothelial cells and fibroblasts were used. Figure 1 is a schematic diagram of the model and test system showing the cultured SIS membrane in a plastic insert.

### Magnetic gradient-forced transport of SPION across the RWM model

The delivery test system in Figure 1 consisted of a 24-cyliner rare earth magnetic array of NdFeB that was placed under a 24 well culture plate where the inserts (Cook Biotech, West Lafayette-IN) fit. The magnetic flux lines created by these magnets change the direction of the magnetization vector of the SPION and force the individual dipole moments of the SPION to align along the flux lines. The magnetic force along the direction of the z-axis forces the SPION to move downward. This force caused the SPION to pass through the RWM model into the culture well below. This magnetic gradient-forced transport (GFT) of SPION is dependent on the magnetic flux density, the magnetic gradient, and the susceptibility of the nanoparticles. The RWM models were exposed to the same magnetic flux density of 0.229 Tesla at a distance of 30 mm from the magnet pole surface as calculated from the gradient plot that we performed. According to our calculations, 10^7 ^SPION crossed the RWM model after 60 minutes of magnetic exposure.

**Figure 2:**
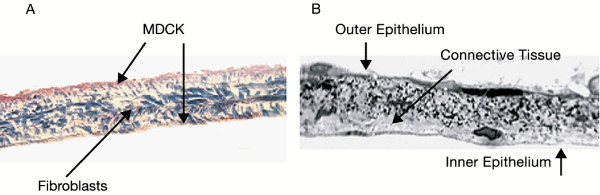
**Histology Section of the RWM Model with 3 Cell Layers and the Rat RWM**. A Histological section of the A. 3 cell-layer RWM model and B. rat RWM. Notice the similarity between the one-cell thick outer and inner epithelial layers on both sides of the SIS membrane as well as in the rat RWM. Swiss 3T3 cells are available both in the connective tissue in the rat RWM as well as within the SIS in the RWM model along with the collagen fibers.

Figure 2A shows a histological section of the SIS membrane seeded with two layers of MDCK cells on both sides and one layer of fibroblasts sandwiched in between. Figure 2B shows a histological section of a rat RWM exhibiting the normal three layer cellular morphology.

### Histology

**Figure 3:**
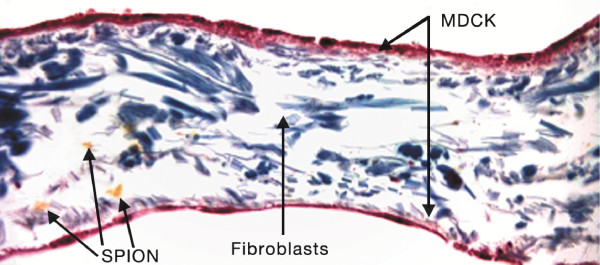
**Histology Section of the RWM Model with SPION**. Histology of the RWM cell culture model. The section shows the outer and inner MDCK epithelial layers. In between the 2 MDCK layers are human fibroblasts. The image also shows clusters of SPION being pulled through with a magnetic field.

**Figure 4:**
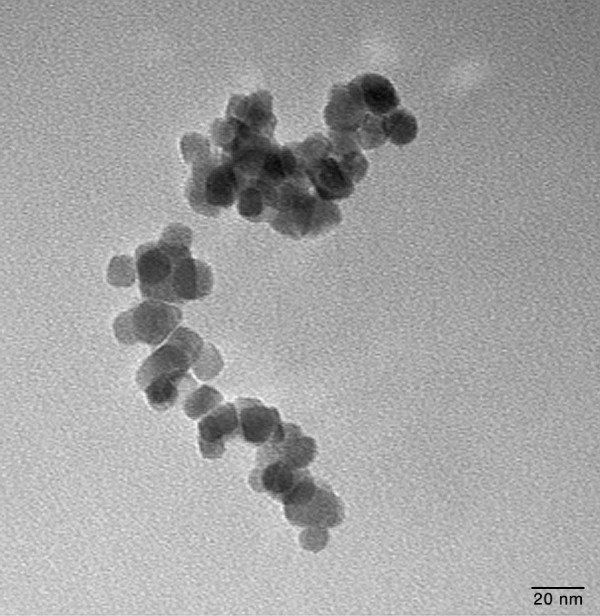
**TEM of the SPION**. A TEM of a sample of the SPION after being pulled through across the RWM model with three cells layers. Shown are clusters of individual SPION particles. Magnification is × 150,000. The scale bar is 20 nm.

Histological sections under light microscope showed the different locations of aggregates of SPION across the RWM model over time. Figure 3 shows SPION aggregates dispersed throughout the RWM model. Histological sections were used to verify that the SPION were non-toxic. Although aggregates of SPION can be seen on and within the SIS membrane at some points, the samples that were collected at the bottom of the culture wells were further analyzed using TEM as shown in Figure 4 and confirmed that single SPION (invisible to light microscopy) were able to pass through the membrane.

**Figure 5:**
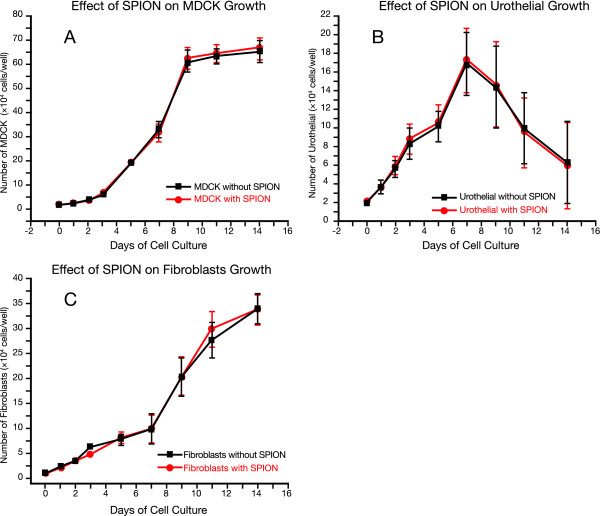
**Effect of SPION on Cell Growth and Proliferation**. Toxicity studies on the biocompatibility of SPION were performed on cells in culture for a period of 14 days. There is no significant difference between cells growing with SPION and cells growing without SPION as shown for A MDCK cells, B. Urothelial cells, and C. fibroblast. Figure 5A. was reprinted from Kopke RD, Wassel RA, Mondalek F, Grady B, Chen K, Liu J, Gibson D, Dormer KJ: *Audiol Neurotol *2006;**11**:123–133, with permission from S. Karger AG, Basel.

### Effect of SPION on cell growth and proliferation

MDCK epithelial cells were cultured in 2, 24-well plates: one plate contained MDCK cells alone while the other plate had MDCK cells cultured with magnetic nanoparticles labelled Nanomag-D NH_2_. Cells were counted on days 1, 2, 3, 5, 7, 9, 11, and 14. Similar experiments were done on 3T3 fibroblasts and human bladder urothelial cells. Figure 5 shows the seeding densities on different days of A. MDCK cells, B. urothelial cells, and C. fibroblasts.

### Transepithelial electrical resistance

**Figure 6:**
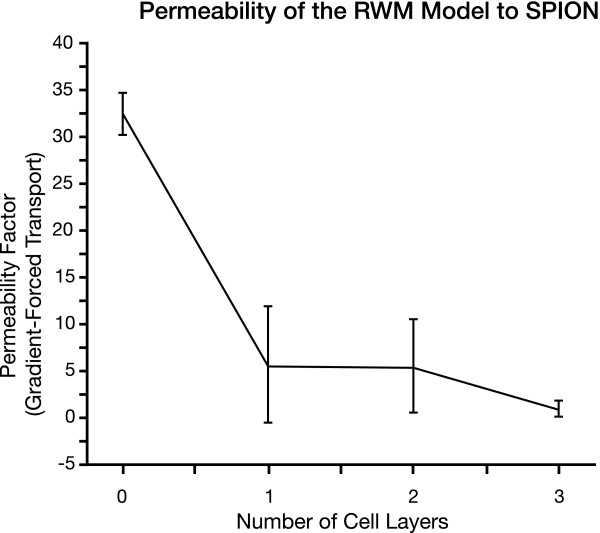
**Permeability of the RWM Model to SPION**. Permeability studies were done on the RWM model with one, two, or three layers of cells. The permeability dropped significantly from the control with no cell layers (SIS alone) to the RWM model with one layer of cells. The permeability did not change much between the one-layer and the two-layer RWM model, but dropped for the three-layer RWM model.

**Figure 7:**
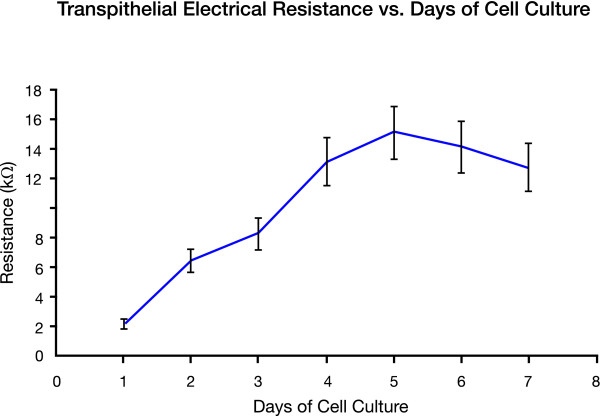
**Transepithelial Electrical Resistance**. The resistance of MDCK cells cultured on the SIS membrane was measured for a period of seven days. The resisitance increased significantly from day 1 to day 4. After day 4, resistance stabilized and changes were no longer significant. The data confirmed that MDCK cells were at or near confluence on day 4.

Cell confluence is a critical and important characterization of the RWM model. Tight junctions must exist between all epithelial cells in order to form a tight seal or obstacle so that magnetic SPION, once pulled through to the other side of the co-cultured SIS membrane, must travel through the co-cultured cells and not through gaps between the cells due to insufficient confluence. The permeability of the RWM model with different cell layers to SPION was calculated as shown in Figure 6. Figure 7 gives the resistance of the MDCK cells over a period of 7 days.

## Discussion

All stages of the experiments showed that the RWM model serves as an *in vitro *human round window membrane, penetrable to SPION by using external magnetic forces. We have demonstrated the use of the magnetic nanoparticles as potential molecules for gene or drug delivery because of their ability to cross the RWM model. This study suggests that cluster-type aggregates of 10 nm magnetic nanoparticles, 130 nm in diameter, can be considered as possible alternatives to viral vectors for gene therapy. Experiments have also shown that they are biocompatible. Toxicity studies confirmed that there was no significant effect on the growth and proliferation of cells in culture. SPION crossed membranes and tissues faster than regular diffusion due to the effect of the external magnetic field.

Our RWM cell culture model is similar to the actual RWM as shown in the histology images comparing the two. Even though the middle layer in the RWM model lacks blood and lymph vessels that are found in a real RWM, the 3 cell layers are similar in their type and function. The toxicity studies of the SPION on cells cultured in dishes showed that there was no significant difference on the seeding density between cells growing with and without SPION, verifying that the SPION are biocompatible. TEER confirmed that cells were at or near cell confluence on day 4 and since all magnetic forced transport experiments were done at day 5 of cell culture; experiments were performed on confluent cells in culture.

A monolayer of MDCK cells forms tight junctions that do not allow even water to go through [24]. In fact, the permeability of the MDCK cells monolayer is so low that it has been studied as a barrier model [25]. This confirms that magnetically-enhanced transportation of the SPION did not occur through MDCK pores, but rather transepithelially through the cells.

The efficiency of this magnetic delivery system using the RWM model described thus far was determined to be 0.02%. Out of the 200 μL of SPION delivered, only 40 nL actually crossed the RWM model under the influence of the external magnetic field. Even though this appears to be an extremely small efficiency, especially in the context of gene transfection using this delivery system, the absolute number of individual SPION pulled through the cells is promising. Using the ratio of SPION to the Alexa Fluor 647 conjugated to the SPION, and from the calibration curve for that dye that was obtained using a SLM 8100 photon-counting spectrofluorometer with a double monochromator in the excitation light path, it was calculated that about 10^7 ^SPION crossed the RWM model after 60 minutes of magnetic exposure.

Due to the limits of detection for the SPION, the time at which the first SPION crosses the RWM model has not yet been determined. The gradient-forced transport of the RWM model with one, two, or three co-cultured cell layers to magnetic SPION labelled Nanomag-D NH_2 _was tested at day 5 of the cell culture for 2 hours at 20-minute intervals using an external magnetic field. Relative concentrations of SPION to cross the RWM model increased exponentially with time, consistent with pure first-order kinetics. The experimental data were analyzed by a non-linear least squares fit to the equation [26].

*C *= *C*_∞ _(1 - *e*^-*kt*^)     **Equation 1**

Where C and C_∞ _are the relative concentrations of the SPION at time= t and ∞ respectively, and k is the first-order rate constant. The parameters C_∞ _and k were used to construct a best-fit curve. The agreement between the theoretical results and the experimental points supports the fact that the transport was first – order, where p < 0.001. The initial slope of each curve (1, 2, or 3 layers) is equal to the permeability factor (Pe). In other words:



Figure 6 shows the different permeability factors of the RWM model cultured with one layer, two layers, and three layers of cells. The gradient-forced transport of the RWM model decreases with increasing layers of cultured cells.

The zero cell-layer model in Figure 6 acted as a control, and consisted of the SIS membrane in the absence of additional cells. The 1 and 2 cell-layer models involved culturing MDCK cells on one side or both sides of the SIS, respectively. The 3 cell-layer model had MDCK cells on both sides of the SIS membrane and a layer of fibroblasts sandwiched between the two cell layers. It is evident that the addition of one cell layer to the SIS membrane significantly decreased the permeability of the model to the SPION. We believe that the permeability to SPION did not change much between the one and two-layer models because the same type of cells were used for each layer and we are studying permeability under the influence of a magnetic force, not gravitational force. However, to develop a more complete understanding of permeability, it would be beneficial to use different cells types with 2 cell-layer model in future studies. For the 3 cell-layer model, fibroblasts were cultured on top of the SIS and allowed to penetrate the membrane for 2 days before were MDCK cells were cultured on top of them. We speculate that the migration of the SWISS 3T3 fibroblasts into the SIS membrane somehow changed the localization of the pores in the SIS, possibly plugging some of the pores, resulting in the steep drop in permeability to SPION as shown in Figure 6.

The TEER was measured for one cell layer of MDCK cells over a period of 7 days. Since the inner and outer epithelial layers are confluent and exhibit very tight junctions in the human RWM, we tested cell confluence to make sure that the SPION would not be able to go through gaps between non-confluent cells. We were not interested in the confluence of fibroblasts because they are not confluent in the actual RWM.

It is necessary to differentiate between diffusion-enhanced permeability and magnetic enhanced permeability, as is the case in this study. Since this is the first time magnetic-induced permeability has been explored using a membrane model, more studies should be conducted in the future to compare the permeability of the RWM model with an actual RWM. This would facilitate the study of both magnetic- and diffusion-enhanced permeability of a known therapeutic molecule. Using the thickness of the magnets (6.35 mm) and the remanance magnetization (1.4 T), it is possible to calculate the strength and gradient of the magnetic field generated. We have preliminary data (Cartwright et al, unpublished data) to calculate the magnetic force on the individual SPION.

It should be noted here that although animal explants of the RWM have been used to establish an *in vitro *model very similar to the actual RWM [18,27,28], the RWM from guinea pigs and rats are only 1 mm in diameter, making SPION detection and quantification a difficult task. The advantage of our cell culture RWM model over animal explants models for evaluating the magnetic-enhanced transport of SPION is ease of detection and quantification of results.

## Conclusion

A tripartite RWM model similar to the human round window membrane has been developed to study the feasibility of transporting magnetic SPION across the RWM. SPION have been magnetically transported and the model was used to compute the efficiency of the magnetic delivery system. This system can be used for quantitative evaluation of the capability for drug/gene delivery through the RWM and is suitable for investigation of putative therapeutic agents in prospective treatments of inner ear diseases.

The RWM model, comprised of a tricellular membrane on a collagen matrix is a novel *in vitro *system for testing the magnetic transport of various biological molecules through the human round window membrane. Targeted magnetic delivery to the inner ear may facilitate minimally invasive targeted delivery of therapeutic agents.

## Methods

### Materials

Culture media reagents were purchased from Gibco-Invitrogen and included: Dulbecco's Modified Eagle Medium (DMEM) [catalogue #11965-092], Fetal Bovine Serum (FBS) [catalogue #16141-079], Keratinocyte Serum Free Media (KSFM) [catalogue # 10724-011], Bovine Pituitary Extract (BPE) [catalogue #13028-14], and Epidermal Growth Factor (EGF) Human Recombinant [catalogue #10450-013].

### Cell culture

MDCK cells were generously provided by Dr. Leo Tsiokas, Department of Cell Biology at the University of Oklahoma Health Sciences Center [OUHSC]. MDCK cells were used between passages 16 and 37, Urothelial cells between passages 3 and 12 and 3T3 cells between passages 7 and 32. Cells were cultured in 100 mm^2 ^culture dishes in DMEM supplemented with 10% FBS for MDCK and 3T3 cells and in KSFM supplemented with 25 mg BPE and 2.5 μg EGF Human Recombinant for urothelial cells. Cells were maintained at 37°C under 5% CO_2_. The medium was changed every other day until the cells reached confluence. Cells were then washed with PBS and detached using Trypsin-EDTA and then cultured on the SIS membrane in plastic inserts that fit the 24 well culture plates. Media was changed on the inserts every 2 days. Cells that were not used for experimentation were cultured in 100 mm^2 ^culture dishes and reincubated at 37°C under 5% CO_2_.

### Transepithelial electrical resistance

Transepithelial electrical resistance (TEER) was measured to confirm the confluence of epithelial cells. The resistance of the cultured SIS membrane was measured using an epithelial volt-ohmmeter (EVOM, World Precision Instruments, New Haven, CT). TEER was determined by applying a square wave alternating current of ± 20 μA at 12.5 Hz with a silver electrode and measuring the potential difference with a silver/silver chloride electrode using EVOM at 37°C in tissue culture media (DMEM with 10% FBS and 1% PS). The seeding density was 4.75 × 10^5 ^cells/ cm^2^.

### Magnetic gradient-forced transport across the RWM model

Due to the fact that the external magnetic field applied to the RWM model to pull the SPION is large enough to overcome the other forces acting on the SPION (like the gravitational force and the drag force), the magnetic forced-gradient transport (GFT) was the only force taken into consideration.

MDCK cells were first seeded on the serosal side of the SIS membrane at a seeding density of 4.4 × 10^5 ^cells/cm^2^. Swiss 3T3 fibroblasts were seeded on the mucosal side of the SIS membrane at a seeding density of 1.8 × 10^3 ^cells/ cm^2^. The fibroblasts were allowed 2 days to penetrate into the SIS membrane. Then MDCK cells were cultured on the mucosal side of the SIS membrane at a seeding density of 4.4 × 10^5 ^cells/cm^2^. SPION labelled Nanomag-D NH_2 _were used in the experiments. All experiments were done at day five of cell culture of the last layer of cells cultured on the RWM model. The magnetic cylinders used (MagStar Technologies) were 6.35 × 6.35 mm and the centers of adjacent magnets were 2 cm apart. A plastic molding (12.8 × 8.6 × 3.1 cm) held the magnets directly under a 24-well culture plate. The magnetic flux density was measured using a Gauss meter Model 5080 (SYPRIS, Orlando-Fl). Sigma plot was used for all statistical calculations

### Histology

After 60 minutes of magnetic exposure, the cells were fixed with fresh made 4% paraformaldehyde for 24 hours. After the cells were fixed, they were put in 3% agar and stored in 10% formalin where they were sectioned on a microtome for microscopic examination. Membrane sections 4–5 μm in thickness were stained with Masson's trichrome. For the rat RWM tissue, we used 2.5% glutaraldehyde for fixation and post fixed in 1% OSO_4 _and stained with uranyl acetate and bismuth oxynitrate for TEM.

### Quantification of GFT

SPION were conjugated with Alexa Fluor 647 (Molecular Probes, Carlsbad-CA). The ratio of SPION to the Alexa Fluor 647 conjugated to the SPION was calculated during the conjugation process and came out to be 1:617 (SPION:Alexa Fluor 647). A calibration curve for different concentrations of the dye was plotted (data not shown); the intensity of the dye was measured using a SLM 8100 photon-counting spectrofluorometer with a double monochromator in the excitation light path.

## Competing interests

The author(s) declare that they have no competing interests.

## Authors' contributions

FGM: did most of the experiments and data analysis. YZ and XG coordinated some experiments. BK, RDK, XG, RLD and KJD helped in drafting the manuscript. All authors read and approved the final manuscript.
